# Targeted Superior Vena Cava Isolation Combined With Pulmonary Vein Isolation Versus Pulmonary Vein Isolation Alone Using Cryoballoon Ablation for Atrial Fibrillation

**DOI:** 10.1002/clc.70469

**Published:** 2026-09-08

**Authors:** Yanxin Han, Yangyang Bao, Yun Xie, Yue Wei, Qingzhi Luo, Tianyou Ling, Wenqi Pan, Ning Zhang, Qi Jin, Changjian Lin, Liqun Wu

**Affiliations:** ^1^ Department of Cardiovascular Medicine Shanghai Ruijin Hospital, Shanghai Jiaotong University School of Medicine Shanghai China

**Keywords:** atrial fibrillation, cryoballoon ablation, non‐pulmonary vein triggers, superior vena cava, targeted ablation

## Abstract

**Background:**

The superior vena cava (SVC) is a common non‐pulmonary vein (non‐PV) trigger site in atrial fibrillation (AF), but its triggers are frequently underdetected without systematic mapping and provocation maneuvers. This study aimed to assess the temporal trend in SVC trigger detection rates and compare the efficacy and safety of cryoballoon‐based targeted SVC isolation combined with pulmonary vein isolation (PVI) versus PVI alone.

**Methods:**

A retrospective analysis was conducted on 990 consecutive patients who underwent cryoballoon ablation for AF between January 2020 and December 2024. Annual SVC trigger detection rates were assessed using the Cochran‐Armitage trend test. After excluding redo cases and performing 1:1 propensity score matching, 55 matched pairs were compared in terms of procedural outcomes, complications, and 12‐month freedom from atrial tachyarrhythmias.

**Results:**

SVC trigger detection rates increased significantly over the study period, from 6.8% in 2020 to 21.3% in 2024 (P for trend = 0.023). After matching, the PVI + SVCI group showed significantly higher 12‐month freedom from atrial tachyarrhythmias compared to the PVI‐only group (90.9% vs. 76.4%, *p* = 0.037). Procedural time was modestly longer in the PVI + SVCI group (93 ± 26 min vs. 80 ± 20 min, *p* = 0.003), but fluoroscopy time (12 ± 5 min vs. 10 ± 4 min, *p* = 0.276) and complication rates (5.5% vs. 3.6%, *p* = 0.500) were comparable between groups, both with low overall complication rates.

**Conclusion:**

Cryoballoon‐based targeted SVC isolation guided by systematic mapping and provocation maneuvers significantly enhances freedom from atrial tachyarrhythmias without compromising safety.

## Graphical abstract

Cryoballoon‐based targeted superior vena cava isolation (SVCI) added to pulmonary vein isolation (PVI) improved 12‐month freedom from atrial tachyarrhythmia compared with PVI alone (90.9% vs. 76.4%, *p* = 0.037) without increasing complications, supporting systematic SVC trigger assessment in atrial fibrillation ablation.

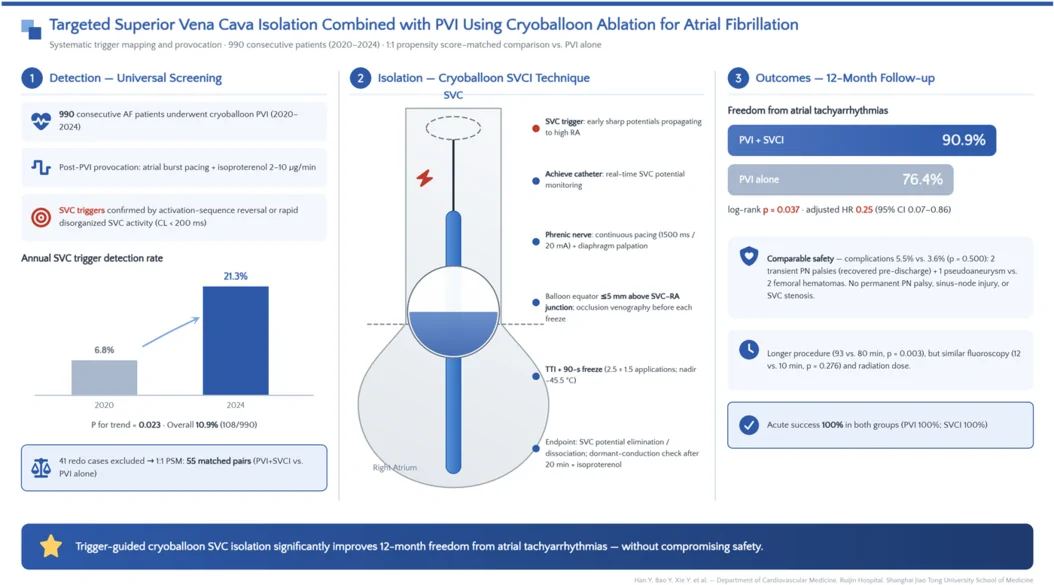

**Abbreviations**
AF: atrial fibrillation
AT: atrial tachyarrhythmias
CBA: cryoballoon ablation
DOAC: direct oral anticoagulants
LAD: left atrial diameter
PAC: premature atrial contraction
PN: phrenic nerve
PV: pulmonary vein
PVI: pulmonary vein isolation
RA: right atrium
SVC: superior vena cava
SVCI: superior vena cava isolation

## 1 Introduction

Atrial fibrillation (AF) is the most prevalent sustained arrhythmia, with pulmonary vein isolation (PVI) serving as the cornerstone of ablation therapy [1, 2]. Despite technological advancements, recurrence rates remain high, largely due to non‐pulmonary vein (non‐PV) triggers [3, 4, 5, 6]. Among these, the superior vena cava (SVC) is the most common non‐PV trigger, responsible for 10%–30% of non‐PV‐related recurrences [7, 8, 9].

Systematic provocation testing and high‐density mapping have demonstrated substantial improvements in trigger detection rates, as undetected intermittent or subtle SVC foci often lead to repeat procedures and compromised long‐term outcomes [10, 11, 12]. An evidence‐based, targeted approach to SVC isolation—performing ablation only for electrophysiologically confirmed triggers—provides a more precise strategy that may improve success rates while minimizing unnecessary lesions and complications [13, 14, 15].

Cryoballoon ablation offers unique advantages for SVC isolation, including the ability to form contiguous lesions and monitor the phrenic nerve in real time, potentially enhancing both safety and efficacy [16, 17, 18]. However, clinical data on cryoballoon‐based targeted SVC isolation combined with PVI are limited. This single‐center retrospective study investigates whether enhanced SVC trigger detection and subsequent targeted isolation, guided by systematic provocation, improve AF ablation outcomes compared to PVI alone.

## 2 Methods

### 2.1 Study Population

This retrospective analysis evaluated 990 consecutive patients undergoing cryoballoon ablation for AF at Shanghai Ruijin Hospital between January 2020 and December 2024. Among them, 108 patients (10.9%) exhibited SVC‐originating triggers during electrophysiological study, including 41 redo ablation cases. To specifically assess the impact of SVC isolation in treatment‐naïve patients, redo cases were excluded, and 1:1 propensity score matching was performed between the PVI + SVCI and PVI‐only groups. Matching covariates included age, sex, BMI, AF type (paroxysmal/persistent), comorbidities, and left atrial diameter, yielding 55 well‐balanced matched pairs. The study protocol was approved by the Institutional Review Board (protocol code RJH201656) and complied with the Declaration of Helsinki. Written informed consent was obtained from all participants prior to the ablation procedure.

### 2.2 Patient Selection Criteria

Baseline clinical characteristics and medical histories, including CHA_2_DS_2_‐VASc scores, were retrospectively extracted from institutional electronic health records. Exclusion criteria included: (1) unmanaged thyroid disorders, (2) intracardiac thrombi identified on preprocedural imaging, (3) advanced valvulopathy requiring intervention, (4) significant coronary artery disease or recent (<3 months) acute coronary syndrome/cardiac surgery, (5) pregnancy, (6) excessive left atrial enlargement (anteroposterior diameter > 50 mm on echocardiography), or (7) additional non‐PV trigger ablation beyond SVC isolation during the index procedure.

### 2.3 Preprocedural Evaluation and Preparation

All enrolled patients underwent comprehensive cardiovascular assessment before ablation. Standard transthoracic echocardiography (TTE) assessed cardiac chamber dimensions, ventricular systolic/diastolic function, and valvular pathology. Three‐dimensional cardiac computed tomography angiography was used for detailed evaluation of left atrial and pulmonary venous anatomy. To ensure procedural safety, transesophageal echocardiography (TEE) was routinely performed within 48 h of the intervention to exclude intracavitary thrombi.

Anticoagulation management adhered to contemporary guidelines. All patients received standard anticoagulation therapy for at least 1 month prior to the procedure, and patients on warfarin maintained an international normalized ratio (INR) of 2–3 before the procedure.

For patients remaining in AF after PVI, electrical cardioversion was performed prior to SVC intervention to optimize procedural conditions.

### 2.4 Cryoablation Procedural Protocol

Ablation procedures were performed under monitored anesthesia care, using intravenous midazolam and fentanyl for conscious sedation. Vascular access was achieved through the right femoral vein, followed by a single transseptal puncture under fluoroscopic guidance to gain left atrial access. Systemic anticoagulation was initiated with an intravenous heparin bolus (100 IU/kg), followed by titration to maintain an activated clotting time greater than 300 s throughout the procedure.

The ablation system utilized a 28‐mm second‐generation cryoballoon catheter (Arctic Front Advance, Medtronic), delivered via a dedicated steerable sheath (FlexCath Advance, Medtronic). Electroanatomical mapping was conducted using the Abbott mapping system, in combination with an intraluminal circular mapping catheter (Achieve, Medtronic), which was sequentially positioned at each pulmonary vein ostium to record baseline electrical activity. Balloon positioning and occlusion were confirmed with selective contrast angiography, with optimal seal defined by complete contrast retention without atrial reflux.

The ablation sequence followed a standardized pattern, starting from the left superior to left inferior pulmonary veins, then progressing to the right superior and right inferior pulmonary veins. Each vein received two cryothermal applications, each lasting 180 s, with real‐time temperature monitoring. Procedural endpoints were defined as complete elimination of pulmonary vein potentials or their electrical dissociation from atrial activity.

Application times and the need for additional freezing were determined at the operator's discretion. As a precaution, cryoenergy delivery was immediately stopped if the balloon temperature reached ‐55°C or lower to prevent excessive tissue injury.

### 2.5 Electrophysiological Characterization and Mapping of SVC Triggers

After PVI, systematic mapping and provocation maneuvers were employed to identify non‐PV triggers in each patient. The stimulation protocol consisted of atrial burst pacing (cycle length 180–300 ms) and pharmacological challenge using isoproterenol infusion (2–10 μg/min). In cases where AF persisted post‐PVI, direct current cardioversion was performed to restore sinus rhythm. If AF reinitiated after cardioversion, pacing, or isoproterenol administration, detailed activation mapping was conducted. Trigger localization was achieved through 12‐lead surface ECG analysis of initiating ectopic beats and intracardiac electrogram correlation.

**Figure 1:**
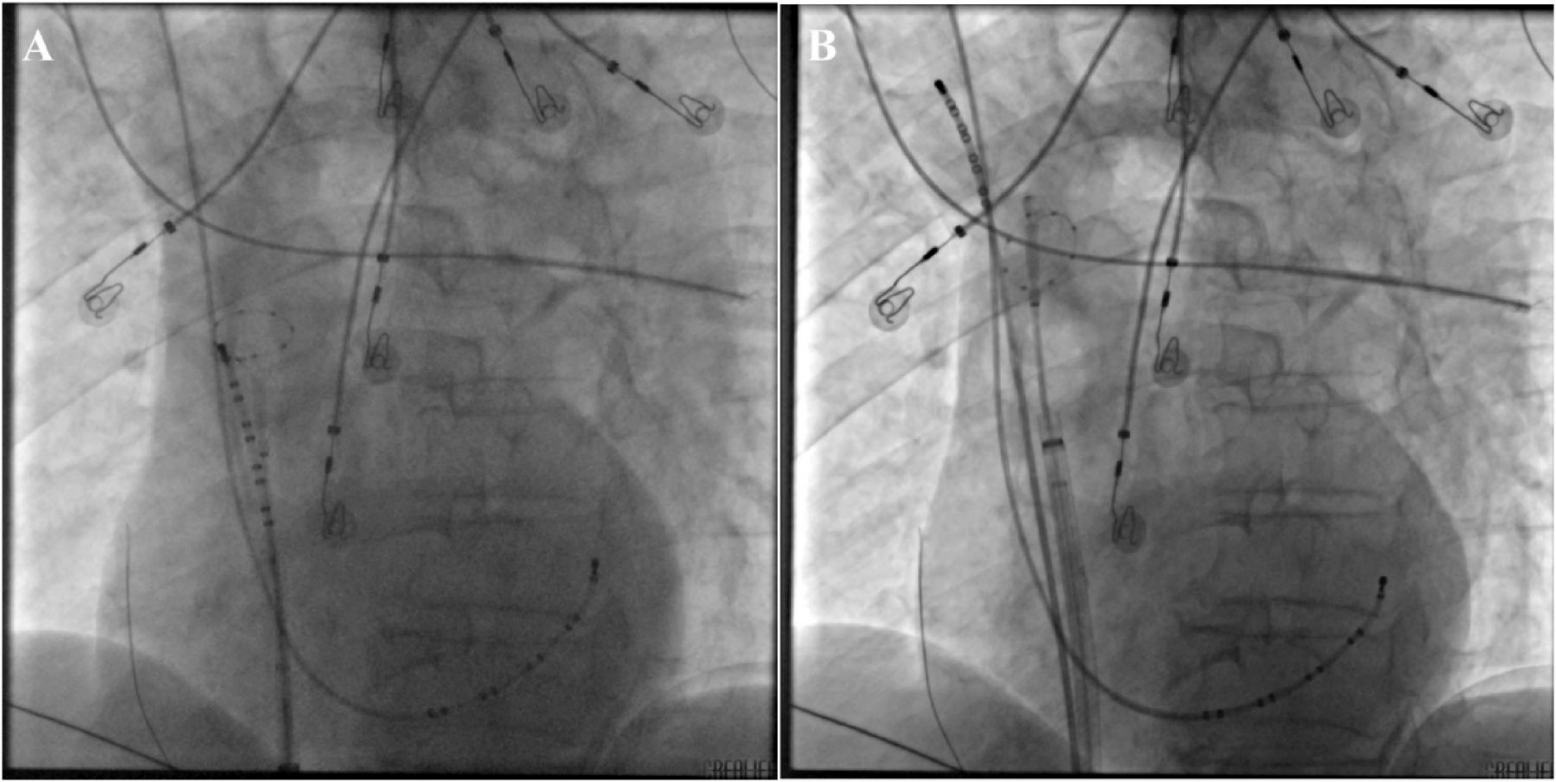
Fluoroscopic images of SVC mapping and cryoballoon ablation. (A) SVC mapping. (B) Cryoballoon ablation with confirmation of total SVC occlusion via contrast venography.

**Figure 2:**
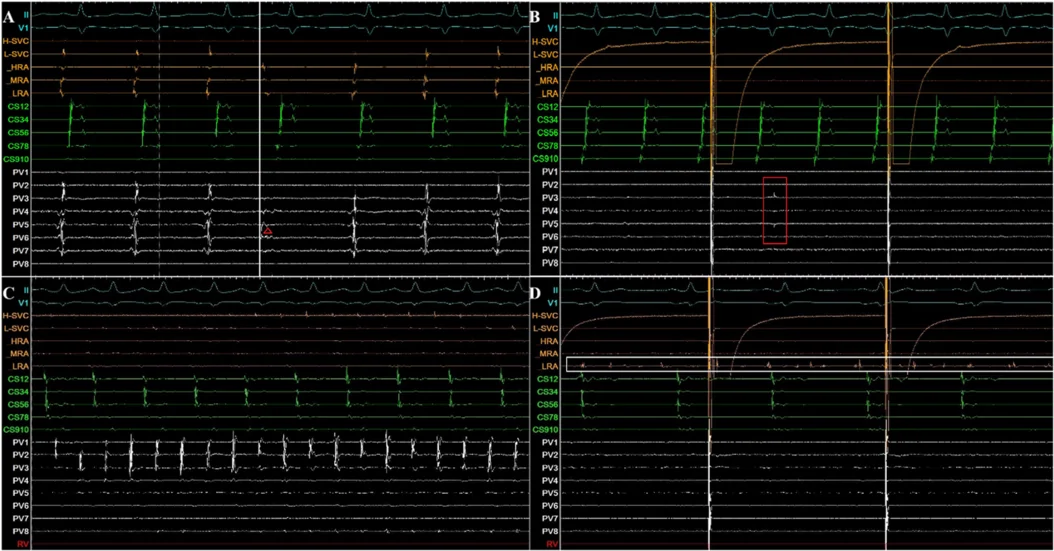
The role of the superior vena cava in atrial fibrillation: spontaneous ectopy, fibrillatory discharge, and electrical isolation from two patients. (A) A single atrial premature contraction resulting from a spontaneous ectopic beat within the SVC. A sharp potential (triangle) recorded via the Achieve catheter marked the earliest activation site during firing in this SVC. This activation subsequently propagated from the SVC to the high right atrium, as indicated by the decapolar catheter positioned in the SVC. (B) Following SVC isolation, spontaneous electrical activity persisted within the SVC, as shown within the rectangular box. (C) High‐frequency activations were observed in the SVC, concurrent with relatively organized activity in the coronary sinus. (D) During cryoablation of the superior vena cava, atrial fibrillation terminated, and sinus rhythm was restored. Notably, high‐frequency activations (within the rectangular box) persisted on the steerable decapolar mapping catheter in the SVC, exhibiting no conduction to the atria.

SVC‐related triggers were initially detected using a deflectable decapolar catheter positioned within the SVC, followed by high‐density mapping with a circular mapping catheter (Achieve, Medtronic) (Figure 1A). The characteristic electrophysiological signature included early, sharp potentials within the SVC region, which showed progressive conduction to the high right atrium (Figure 2A). Diagnostic confirmation required meeting one of two criteria: (1) inversion of the normal activation sequence during arrhythmia initiation, where far‐field atrial electrograms preceded near‐field SVC potentials in sinus rhythm, but reversed timing was observed during trigger onset, or (2) the presence of rapid, disorganized electrical activity within the SVC (cycle length < 200 ms), concurrent with relatively organized activation patterns in the coronary sinus. All identified SVC triggers were retrospectively re‐adjudicated using the final unified diagnostic criteria, regardless of the year of procedure, to ensure consistency of exposure definition across the study period. These distinct electrophysiological patterns allowed reliable differentiation between true SVC triggers and passive conduction phenomena (Figure 2C).

### 2.6 SVC Isolation Technique

Cryoablation for SVC isolation was performed only in patients with identified SVC triggers, following a standardized protocol described in our previous study. Initially, the cryoballoon was inflated in the right atrium and carefully maneuvered toward the SVC‐right atrium (SVC‐RA) junction. Complete vessel occlusion was confirmed through contrast angiography, demonstrating full contrast retention within the SVC without atrial reflux. This step precisely delineated the anatomical transition between the SVC and right atrium. Following deflation, the cryoballoon was advanced into the SVC and reinflated. The ablation zone was strategically positioned with the cryoballoon's equatorial band slightly superior to the SVC‐RA junction. Anatomic verification was performed using a deflectable decapolar catheter (2‐5‐2 mm spacing) as a spatial reference, ensuring the ablation site remained within 5 mm of the junction. The Achieve catheter was repositioned proximally to monitor SVC potentials in real time. Pre‐ablation venography was performed to reconfirm complete SVC occlusion before each application (Figure 1B). Electrical isolation was defined by the complete disappearance of SVC potentials or their dissociation from right atrial activity (Figures 2B,D). The standardized ablation protocol involved a 90‐second cryoapplication following time‐to‐isolation. Energy delivery was immediately discontinued if any of the following occurred: loss of phrenic nerve capture or reduced diaphragmatic excursion, significant bradyarrhythmias, or balloon temperatures below −55°C. The need for additional applications was determined by the operator based on real‐time electrophysiological assessment. After a 20‐min observation period, isoproterenol challenge testing (2–10 μg/min) was administered to assess for dormant conduction. Final confirmation of SVC‐RA entry block was achieved through comprehensive electrophysiological assessment.

All procedures were performed by experienced electrophysiologists (minimum 5 years of experience in AF ablation; collective experience of > 2000 cryoballoon ablation procedures). The two objective electrophysiological criteria for SVC trigger identification—(1) inversion of the normal activation sequence and (2) rapid disorganized SVC activity (cycle length < 200 ms)—were designed to be operator‐independent and were applied consistently throughout the study period. All identified triggers were retrospectively re‐adjudicated using these unified criteria.

### 2.7 Phrenic Nerve Monitoring Protocol

A comprehensive nerve protection strategy was implemented during right‐sided pulmonary vein and SVC ablation procedures. Prior to energy delivery to right pulmonary or SVC structures, a deflectable decapolar catheter was positioned in the SVC to enable phrenic nerve stimulation. Continuous pacing was performed with a 1500‐ms cycle and 20‐mA output. Diaphragmatic contraction was assessed manually by palpating the abdomen during pacing. The phrenic nerve was continuously monitored to minimize the risk of phrenic nerve paralysis.

### 2.8 Postprocedural Monitoring and Outcome Assessment

Following the ablation procedure, comprehensive cardiac monitoring was implemented for all participants through continuous telemetric ECG surveillance during the initial recovery phase. Oral anticoagulant therapy was prescribed for a minimum of 2 months post‐ablation, with subsequent management tailored to individual thromboembolic risk profiles. Structured clinical follow‐up was scheduled at 1, 3, and 6 months post‐procedure, followed by semiannual evaluations thereafter.

Rhythm monitoring primarily involved 24‐h ambulatory Holter recordings obtained at each scheduled follow‐up visit at 1, 3, 6, and 12 months. For patients reporting symptoms indicative of arrhythmia recurrence, prompt electrocardiographic evaluation was recommended at the nearest medical facility. The first 3 months postoperatively were designated as the blanking phase for procedural recovery. Treatment efficacy was determined by the occurrence of electrocardiographically confirmed atrial tachyarrhythmia episodes lasting beyond 30 s after the blanking period.

### 2.9 Statistical Analysis Methods

Continuous variables are presented as mean ± standard deviation or median (interquartile range), as appropriate. Categorical variables are expressed as frequencies and percentages. Differences between groups were assessed using the Student's *t*‐test, Mann–Whitney *U* test, chi‐square test, or Fisher's exact test, as applicable. To assess the temporal trend in SVC trigger detection rates across the study period (2020–2024), the Cochran–Armitage trend test was used. A two‐sided *p* value of <0.05 was considered statistically significant. Time‐to‐event outcomes, specifically freedom from atrial tachyarrhythmias, were analyzed using Kaplan−Meier survival analysis, with statistical significance determined by the log‐rank test. To confirm the robustness of survival findings, a multivariable Cox proportional hazards regression model was constructed, with atrial tachyarrhythmia recurrence as the dependent variable and treatment group, AF type, left atrial diameter, CHA_2_DS_2_‐VASc score, age, and sex as independent variables. All analyses were conducted using R software (version 4.3.0; R Foundation for Statistical Computing) or SPSS (version 26.0; IBM Corp.).

## 3 Results

### 3.1 SVC Triggers Detection Rate

**Figure 3:**
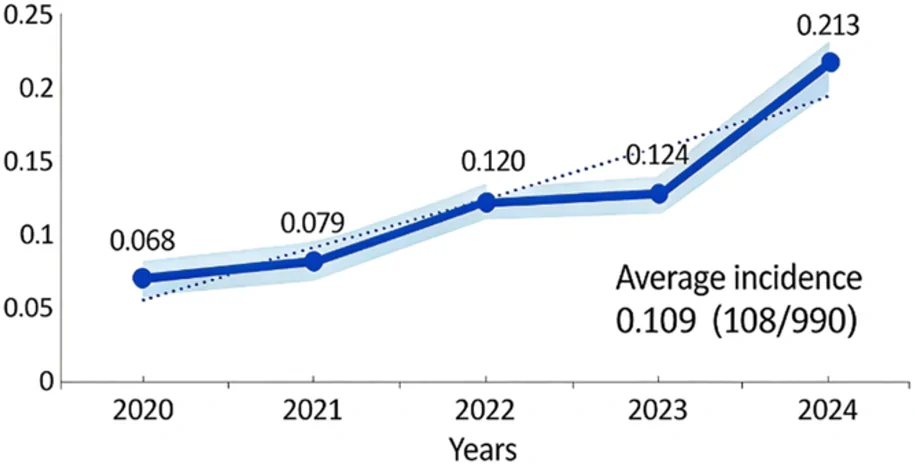
Annual SVC trigger detection rate, 2020–2024.

Over the 5‐year study period (2020–2024), the annual detection rate of SVC‐originating triggers showed a marked and consistent increase, rising from 6.8% in 2020 to 21.3% in 2024 (P for trend = 0.023). The overall detection rate across the entire cohort of 990 patients was 10.9% (108/990) (Figure 3).

### 3.2 Study Population and Baseline Characteristics

A total of 990 consecutive patients undergoing cryoballoon ablation for AF were initially screened. Among them, 108 (10.9%) exhibited SVC‐originating triggers during electrophysiological study. After excluding 41 redo ablation cases and performing 1:1 propensity score matching for treatment‐naïve patients, 55 well‐matched pairs (PVI + SVCI group *vs*. PVI‐only group) were included in the final analysis.

Table 1 presents the baseline clinical characteristics of the matched cohorts, with no significant differences observed between the groups in terms of age, sex, BMI, AF type (paroxysmal/persistent), left atrial diameter, or CHA_2_DS_2_‐VASc score (all *p* > 0.05), confirming successful matching and comparability.

**Table 1:** Baseline characteristics of the population.

	PVI + SVCI	PVI	*p*
Age	62.2 ± 9.0	62.1 ± 9.1	0.966
Sex (female%)	34.5	34.5	1
BMI	23.9 ± 3.4	24.5 ± 2.6	0.288
AF type (PAF%)	80	80	1
LAD (mm)	41.1 ± 4.6	40.9 ± 4.2	0.88
CHA_2_DS_2_‐VASc	2.2 ± 1.3	1.8 ± 1.3	0.163
LVEF	64.4 ± 7.5	64.9 ± 4.6	0.659
LVEDD	49.53 ± 5.37	49.24 ± 4.15	0.751
d‐dimer	0.38 ± 0.44	0.35 ± 0.39	0.676
Hypertension (*n*)	30	30	1
DM	4	4	1
CAD	7	7	1
Smoking	11	11	1
Stroke/TIA	3	3	1

Abbreviations: BMI = body mass index, CAD = coronary artery disease, DM = diabetes mellitus, LVEDD = left ventricular end diastolic diameter, LVEF = left ventricular ejection fraction, TIA = transient ischemic attack.

### 3.3 Procedural Data

The acute procedural success rate for PVI was 100% in both groups. In the PVI + SVCI group, acute electrical isolation of the SVC was achieved in all 55 patients (100%). The mean number of cryoapplications for SVC isolation was 2.5 ± 1.5. Although the addition of SVCI was associated with a longer total procedural time (93 ± 26 min vs. 80 ± 20 min, *p* = 0.003), it did not lead to a significant increase in fluoroscopy exposure (12 ± 5 min vs. 10 ± 4 min, *p* = 0.276).

### 3.4 Primary Efficacy Endpoint: Freedom From AT

**Figure 4:**
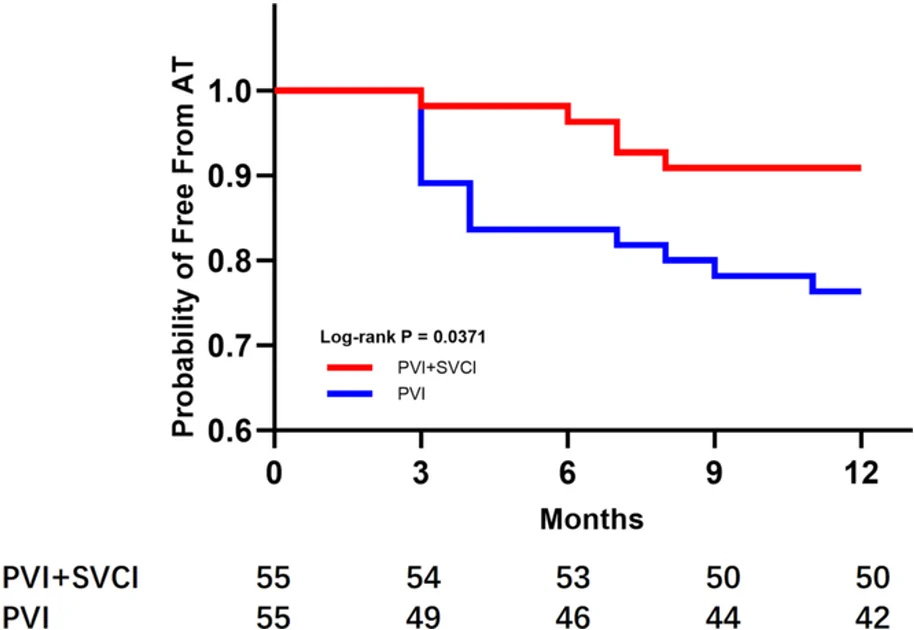
Kaplan−Meier estimates of freedom from any atrial tachyarrhythmia episodes. AT, atrial tachyarrhythmias; PVI, pulmonary vein isolation; SVCI, superior vena cava isolation.

After a 3‐month blanking period, the primary endpoint of atrial tachyarrhythmia recurrence was significantly less frequent in the PVI + SVCI group. Kaplan−Meier survival analysis demonstrated that freedom from AT at 12 months was significantly higher in the PVI + SVCI group compared to the PVI‐only group (90.9% vs. 76.4%, log‐rank *p* = 0.037) (Figure 4).

A multivariable Cox regression model was constructed to identify independent predictors of atrial tachyarrhythmia recurrence. The model included treatment group (PVI + SVCI vs. PVI‐only), AF type (paroxysmal vs. persistent), left atrial diameter, CHA_2_DS_2_‐VASc score, age, and sex. PVI + SVCI remained an independent protective factor against AT recurrence (HR = 0.25, 95% CI: 0.07–0.86, *p* = 0.028), confirming the robustness of the Kaplan‐Meier findings (Supporting Information S1: Table 1).

Sensitivity analyses, including restriction to the later study period (2023–2024), variation of the blanking period, and per‐protocol analysis, yielded consistent results (data not shown).

### 3.5 Safety Endpoint: Complications

The overall complication rate was low and comparable between the two groups. In the PVI + SVCI group, three complications occurred: two cases of transient phrenic nerve palsy that fully recovered before discharge, and one case of femoral pseudoaneurysm. In the PVI‐only group, two complications were observed: two cases of femoral hematoma (5.5% vs. 3.6%, *p* = 0.500). No permanent phrenic nerve palsy, sinoatrial node injury requiring pacemaker implantation, or SVC stenosis occurred in either group.

## 4 Discussion

Our single‐center retrospective study demonstrates that systematic SVC trigger mapping combined with cryoballoon‐based targeted ablation significantly improves outcomes in patients undergoing AF ablation. Key findings include a progressive increase in SVC trigger detection and superior outcomes with targeted SVC isolation.

The steady and significant rise in the SVC trigger detection rate—from 6.8% in 2020 to 21.3% in 2024 (P for trend = 0.023)—should be interpreted not as a shift in disease prevalence, but rather as a direct result of institutional learning and protocol maturation. This trajectory reflects the integration of evolving electrophysiological knowledge into routine clinical practice, captured across three interconnected dimensions of practice change.

1. From Reactive Targeting to Universal Screening

Initially, SVC mapping was selectively performed, often triggered by clinical suspicion such as the absence of PV reconnection during redo procedures, specific P‐wave morphologies on surface ECG, or spontaneous ectopy observed during post‐PVI observation. This reactive approach, while intuitive, systematically under‐assessed intermittent SVC triggers or those masked by ongoing AF. Over the course of the study, we transitioned to a universal screening model, in which systematic SVC electrophysiological assessment—incorporating high‐dose isoproterenol provocation and post‐cardioversion re‐induction—became a standard component of all AF ablation procedures, regardless of baseline suspicion. This shift from “when indicated” to “unless omitted” substantially expanded the diagnostic denominator and was the primary driver of the observed detection trend.

2. Refinement of Diagnostic Thresholds

Alongside the expansion of screening, the electrophysiological definition of clinically actionable SVC triggers was refined. Initially, we adhered to a strict, event‐based criterion: only SVC ectopic activity that directly initiated AF was considered a valid trigger for isolation. This high‐threshold approach, while highly specific, lacked sensitivity to sub‐sustaining but arrhythmogenic substrates—such as SVC‐originating premature atrial contractions (PACs) or non‐sustained atrial tachycardia, which, while not immediately degenerating into AF, represented ectopic foci that propagated to the right atrium.

Over time, informed by accumulating evidence and increasing operator experience, we adopted a broader, electrogram‐based diagnostic framework. In this refined paradigm, demonstrating the earliest activation within the SVC with propagation to the high right atrium, evidenced by the reversal of the typical far‐field/near‐field potential sequence, became sufficient diagnostic evidence for an SVC trigger, regardless of whether the initiating beat led to sustained AF. This shift in diagnostic strategy—from “proven culprit” to “confirmed source”—enhanced sensitivity without sacrificing specificity, as the electrogram reversal remains a robust, operator‐independent hallmark of true SVC origin [7].

3. Lowering of the Interventional Threshold

Advances in detection and diagnosis have been accompanied by a progressive decline in the therapeutic threshold for SVC isolation. Initially, even when SVC ectopy was documented, a conservative observational approach was often chosen, particularly for isolated PACs without sustained arrhythmia induction. This hesitation stemmed from uncertainty regarding the clinical significance of such findings and concerns about procedural safety, particularly the risks of sinus node injury and phrenic nerve damage.

As cumulative experience—supported by our own evolving safety data—demonstrated that cryoballoon‐based SVC isolation could be performed with high efficacy and low complication risk, the clinical perspective shifted. By the later phase of the study, the presence of electrophysiologically confirmed SVC‐originating activity, regardless of its sustainability, became a sufficient indication for isolation. This lowered interventional threshold directly contributed to the rising detection rate, as more patients with diagnostically confirmed SVC foci proceeded to ablation and were thus included in the SVC‐positive cohort.

These three concurrent trends—the universalization of screening, liberalization of diagnostic criteria, and lowering of the interventional threshold—converged to explain the striking temporal trend observed in our data. Importantly, the underlying disease did not change; rather, our ability to recognize and address it improved. The 2024 detection rate of 21.3%, derived from the most mature phase of our learning curve, likely represents the closest approximation to the true prevalence of electrophysiologically active, clinically actionable SVC foci in a consecutive AF ablation cohort. This figure aligns closely with the upper range of detection rates reported in studies employing systematic, high‐intensity provocation protocols (10%–30%), suggesting that our current practice has achieved diagnostic parity with specialized academic centers [9, 19, 20].

This trajectory carries a critical implication: low institutional detection rates should not be regarded as immutable baselines [11]. Instead, they should prompt a thorough reassessment of screening protocols, diagnostic criteria, and interventional thresholds. SVC triggers are often underdetected during standard ablation, leading to incomplete treatment and higher recurrence rates [6, 21]. Our experience illustrates that diagnostic yield can be improved through deliberate evolution of practice, and that the true prevalence of SVC triggers may be significantly higher than previously estimated in historical studies [7, 22].

Patients undergoing PVI combined with cryoballoon‐guided SVC isolation exhibited significantly higher 1‐year freedom from AF/AT compared to those who received PVI alone (90.9% vs. 76.4%, *p* = 0.037). This finding is consistent with recent evidence suggesting that selective SVC isolation—when guided by trigger mapping—enhances long‐term success, while empirical SVC isolation offers no clear benefit [23, 24, 25, 26]. The cryoballoon's ability to achieve durable SVC isolation with contiguous lesions, alongside real‐time phrenic nerve monitoring, likely contributed to both efficacy and safety.

Notably, our success rates surpassed those of historical radiofrequency‐based SVC isolation cohorts, possibly due to the more consistent lesion durability achieved with cryoenergy [27, 28]. No patient in our cohort developed inappropriate sinus tachycardia (IST) following SVCI. This may be attributable to the precise positioning of the cryoballoon at the SVC–RA junction, guided by contrast venography, which minimizes injury to the sinus node complex and its autonomic innervation at the crista terminalis. IST has been reported as a rare complication following combined pulmonary vein and SVC isolation with cryoballoon ablation [29], underscoring the importance of meticulous anatomical guidance during SVCI. Early experience further supports the feasibility of cryoballoon‐based SVC isolation [30]. In our series, continuous heart rate monitoring during cryoapplication and immediate termination upon detection of significant bradyarrhythmias provided additional protection against sinus node injury.

Additionally, the presence of persistent left SVC (PLSVC), the most common congenital thoracic venous anomaly (prevalence ~0.3%–0.5%), warrants consideration. PLSVC can harbor arrhythmogenic musculature and has been associated with multiple types of atrial arrhythmia [31]. In our cohort, no cases of PLSVC were identified on pre‐procedural 3D cardiac CTA; however, systematic screening for this anomaly was not the primary focus of this study. Centers performing SVC trigger mapping should be aware of this potential substrate, and isolation of a persistent LSVC should be considered when arrhythmogenic activity is documented (Table 2).

**Table 2:** Procedural details.

	PVI + SVCI	PVI‐only	*p*
SVC diameter, mm	21 ± 1.0		
Mean number of applications, *n*	2.5 ± 1.5		
SVC freezing time, s	223.81 ± 141.54		
Time to isolation (s)	28.14 ± 14.86		
Temperature at isolation, °C	−28.17 ± 7.17		
Nadir temperature, °C	−45.54 ± 6.09		
Mean total procedural time, min	93.33 ± 25.78	79.94 ± 19.73	0.003
Mean fluoroscopy time, min	11.69 ± 4.61	10.13 ± 3.65	0.276
Average radiation dose, μGycm^2^	1560–3192	1790–4094	0.295
Average radiation dose, mGy	86.5–193.6	108.2–253.8	0.246
Left superior PV			
Cryoapplication duration per vein, s	353.16 ± 110.92	357.41 ± 115.71	0.847
Cryoapplication frequency per vein	2.47 ± 0.93	2.40 ± 0.71	0.636
Nadir temperature, °C	−49.67 ± 4.97	−48.93 ± 6.80	0.488
Left inferior PV			
Cryoapplication duration per vein, s	317.51 ± 82.08	321.34 ± 88.54	0.818
Cryoapplication frequency per vein	2.11 ± 0.56	2.23 ± 0.72	0.350
Nadir temperature, °C	−44.5 ± 4.30	−44.3 ± 5.29	0.824
Right superior PV			
Cryoapplication duration per vein, s	316.44 ± 69.44	322.30 ± 83.50	0.694
Cryoapplication frequency per vein	2.31 ± 0.60	2.35 ± 0.70	0.734
Nadir temperature, °C	−51.38 ± 4.82	−50.13 ± 5.33	0.205
Right inferior PV			
Cryoapplication duration per vein, s	369.76 ± 110.78	369.80 ± 136.26	0.999
Cryoapplication frequency per vein	2.56 ± 0.80	2.56 ± 1.05	0.964
Nadir temperature, °C	−46.42 ± 7.03	−45.87 ± 5.97	0.665
Complications	3	2	1
Femoral hematoma	0	2	0.495
Femoral pseudoaneurysm	1	0	1
Right phrenic nerve palsy			
During right PVI	0	0	1
During SVCI	2	0	0.495

## 5 Limitations

This study has several limitations. First, as a single‐center retrospective analysis, inherent selection bias and unmeasured confounding factors cannot be entirely ruled out, despite propensity score matching. The evolving institutional practice over the 5‐year study period introduces temporal bias; although all cases were re‐adjudicated using uniform electrophysiological criteria and sensitivity analyses were performed, residual confounding remains possible. Therefore, the findings may not be generalizable to other populations or practice settings and should be considered hypothesis‐generating. Second, the 5‐year study period included changes in screening protocols, diagnostic criteria, and interventional thresholds. The observed increase in SVC trigger detection rate reflects this composite practice evolution rather than a single factor. While cases were re‐adjudicated using uniform criteria, residual temporal bias remains a possibility. Third, the relatively small sample size after matching (55 pairs) provides adequate statistical power for the primary efficacy endpoint but is inherently underpowered for detecting rare but clinically important safety outcomes such as permanent phrenic nerve injury or sinus node dysfunction. Although safety data from the entire SVCI cohort (*n* = 67) are consistent with published literature, larger prospective studies are needed to definitively establish the safety profile. The 12‐month follow‐up with intermittent 24‐h Holter monitoring may underestimate asymptomatic AF recurrences and bias efficacy estimates upward. Although this limitation applies equally to both groups, future prospective studies should consider continuous cardiac monitoring strategies, such as implantable loop recorders, to more accurately quantify arrhythmia burden. Finally, the findings are specific to a high‐volume tertiary center using second‐generation cryoballoon technology and a highly protocolized screening workflow. While the core elements of the provocation and mapping protocol are transferable to other experienced electrophysiology laboratories, the results may not be directly applicable to lower‐volume centers, different energy sources, or alternative ablation technologies.

## 6 Conclusions

Targeted cryoballoon SVC isolation, guided by systematic provocation, significantly improves ablation outcomes. Our experience further demonstrates that SVC trigger detection rates are modifiable through protocol refinement, revealing a true prevalence higher than historically recognized. Routine systematic SVC assessment should be incorporated into practice to enhance the durability of AF ablation.

## Funding

The authors have nothing to report.

## Disclosure

The authors have nothing to report.

## Ethics Statement

The study was conducted according to the guidelines of the Declaration of Helsinki and approved by the Institutional Review Board of Ruijin Hospital, Shanghai Jiao Tong University School of Medicine (protocol code RJH201656).

## Conflicts of Interest

The authors declare no conflicts of interest.

## Supporting information


**Table S1:** Multivariate Cox regression analysis of freedom from atrial tachycardia recurrence.

## Data Availability

The data that support the findings of this study are available from the corresponding author upon reasonable request.
