# Supplementary material for: Targeted Superior Vena Cava Isolation Combined With Pulmonary Vein Isolation Versus Pulmonary Vein Isolation Alone Using Cryoballoon Ablation for Atrial Fibrillation
Source: Clin Cardiol. 2026 Sep 8;49(9):e70469. doi: 10.1002/clc.70469 (PMC13554712; doi:10.1002/clc.70469)
Supplement: Supplementary file 1 [file CLC-49-e70469-s001.docx]

Table S1 Multivariate Cox regression analysis of freedom from atrial tachycardia recurrence.

|  | HR | 95% CI | *p* |
| --- | --- | --- | --- |
| AF type (persistent) | 2.11 | 0.79-5.63 | 0.135 |
| Left atrial diameter (mm) | 0.95 | 0.86-1.06 | 0.392 |
| CHA2DS2-VASc | 1.07 | 0.75-1.51 | 0.711 |
| Age (years) | 1.03 | 0.98-1.09 | 0.241 |
| Sex (female) | 0.93 | 0.35-2.47 | 0.877 |
| PVI▒+▒SVCI | 0.25 | 0.07-0.86 | 0.028 |
|  | | | |
